# Comparative Evaluation of Clinical and Radiographic Outcomes of Regenerative Endodontic Therapy Using Blood Clot Versus Platelet-Rich Fibrin Scaffolds in Immature Permanent Teeth: A Prospective Cohort Study

**DOI:** 10.7759/cureus.113983

**Published:** 2026-08-05

**Authors:** Swati Sagar, Prachi Joshi, R. S. Senthil Rajan, K. Kasthuripriya, Vigneswaran S., Pallavi Chandrasekhar, Sajidali S Saiyad

**Affiliations:** 1 Department of Dentistry, Darbhanga Medical College and Hospital, Darbhanga, IND; 2 Department of Conservative Dentistry and Endodontics, M. A. Rangoonwala College of Dental Sciences and Research Centre, Pune, IND; 3 Department of Periodontology, RajaRajeswari Dental College and Hospital, Bengaluru, IND; 4 Department of Preventive and Social Medicine, Jawaharlal Institute of Postgraduate Medical Education and Research, Puducherry, IND; 5 Department of Pedodontics and Preventive Dentistry, Priyadarshini Dental College and Hospital, Thiruvallur, IND; 6 Department of Physiology, Pacific Medical College and Hospital, Pacific Medical University, Udaipur, IND

**Keywords:** blood clot scaffold, cone-beam computed tomography, immature permanent teeth, periapical healing, platelet-rich fibrin, pulp necrosis, radiographic root area, regenerative endodontic treatment, root maturation

## Abstract

Background and objective

Regenerative endodontic treatment (RET) promotes continued root development in immature permanent teeth with pulp necrosis; however, the optimal scaffold for maximizing regenerative outcomes remains uncertain. The objective of this study is to compare the clinical and radiographic effectiveness of platelet-rich fibrin (PRF) and conventional blood clot scaffolds using standardized cone-beam computed tomography (CBCT)-based assessment and to identify independent predictors of regenerative success.

Methods

This prospective, non-randomized comparative clinical study was conducted at the Department of Pediatric Dentistry, Pacific Hospital, Udaipur, Rajasthan, India, and included 40 children with non-vital immature permanent maxillary incisors treated with RET using either a blood clot scaffold (n = 20) or a PRF scaffold (n = 20). CBCT-based radiographic analyses were performed at baseline and the 15-month follow-up. The primary outcome was the percentage increase in radiographic root area (RRA), while secondary outcomes included percentage changes in root length, apical diameter, and periapical lesion size. Clinical outcomes were evaluated throughout follow-up. Between-group comparisons were performed using independent t-tests or Mann-Whitney U tests, as appropriate, and multivariable linear regression was used to identify independent predictors of RRA improvement.

Results

Radiographic analysis included 37 teeth (blood clot, n = 18; PRF, n = 19). The PRF group demonstrated a significantly greater increase in RRA than the blood clot group (19.15 ± 6.22% vs. 9.72 ± 5.18%; mean difference, 9.43%; 95% confidence interval (CI), 5.86-13.00; p < 0.001; Cohen's d = 1.58). PRF also achieved significantly greater reductions in apical diameter (p = 0.028) and periapical lesion size (p < 0.001), whereas the increase in root length did not differ significantly between groups (p = 0.415). Both groups exhibited significant within-group radiographic improvement and high clinical success rates (blood clot: 90%; PRF: 95%). Multivariable regression identified PRF as the strongest independent predictor of RRA improvement (β = 9.43, p < 0.001), while larger baseline periapical lesion size was negatively associated with regenerative outcomes.

Conclusions

PRF was associated with significantly greater structural root maturation and periapical healing than the conventional blood clot scaffold while maintaining comparable clinical success. Standardized CBCT-based quantitative assessment supports PRF as an effective scaffold for RET and provides robust evidence to inform clinical decision-making.

## Introduction

Regenerative endodontic treatment (RET) has emerged as a biologically based approach for the management of immature permanent teeth with necrotic pulps by shifting the treatment paradigm from the creation of an artificial apical barrier to regeneration of the pulp-dentin complex. Unlike conventional apexification, which arrests further root development and leaves thin dentinal walls susceptible to fracture, RET may promote continued root development and regeneration of the pulp-dentin complex in immature permanent teeth with pulp necrosis [1,2]. The success of RET relies on the interaction of stem cells, bioactive signaling molecules, and an appropriate scaffold that supports cellular migration, proliferation, angiogenesis, and differentiation [2,3].

The scaffold provides structural support and serves as a reservoir for bioactive molecules essential for tissue repair. Conventionally, induced intracanal bleeding generates a blood clot scaffold [2], as recommended by the American Association of Endodontists clinical protocol [1]; however, blood clot formation is unpredictable, with considerable variability in scaffold volume, stability, and growth factor content. Consequently, autologous platelet concentrates have emerged as promising alternatives because of their enhanced biological properties and sustained release of growth factors [2,4]. Platelet-rich fibrin (PRF), a second-generation platelet concentrate, possesses a dense fibrin matrix enriched with platelets, leukocytes, and growth factors - including platelet-derived growth factor, transforming growth factor-β, and vascular endothelial growth factor - thereby providing a favorable microenvironment for angiogenesis, stem cell recruitment, and hard tissue formation [4]. Additionally, dentin conditioning with ethylenediaminetetraacetic acid releases bioactive dentin matrix proteins that regulate stem cell differentiation and odontoblastic activity, highlighting the biological importance of both scaffold selection and appropriate canal disinfection during regenerative procedures [5].

Recent clinical studies have reported favorable clinical and radiographic outcomes following PRF-assisted regenerative procedures, including enhanced root maturation and periapical healing compared with conventional regenerative protocols [6,7]. Likewise, systematic reviews and meta-analyses have suggested that platelet concentrate scaffolds may improve radiographic root development compared with conventional blood clot scaffolds [8,9]. Nevertheless, the available evidence remains limited by methodological heterogeneity, relatively small sample sizes, inconsistent outcome measures, and limited use of standardized cone-beam computed tomography (CBCT)-based quantitative assessment. CBCT provides three-dimensional, reproducible quantitative evaluation of root morphology and periapical changes, enabling more comprehensive assessment than conventional two-dimensional radiography. Moreover, previous investigations have predominantly evaluated isolated parameters such as root length or apical diameter using two-dimensional imaging, which is susceptible to geometric distortion and cannot fully characterize the three-dimensional nature of root development. Comprehensive assessment of radiographic root area (RRA) - a validated quantitative method integrating changes in both root length and dentinal wall thickness into a single composite measure - has received comparatively less attention, despite its potential to provide a more comprehensive assessment of overall root maturation [10].

We hypothesized that PRF would result in significantly greater radiographic root maturation than the conventional blood clot scaffold.

Therefore, the primary objective of the present study was to compare the percentage increase in RRA following RET using PRF and conventional blood clot scaffolds in immature permanent teeth with pulp necrosis. The secondary objectives were to compare changes in root length, apical diameter, and periapical lesion size; evaluate clinical and radiographic success rates; assess longitudinal clinical outcomes during follow-up; and identify independent predictors of radiographic root development using multivariable regression analysis. This study provides standardized CBCT-based quantitative evidence regarding the comparative effectiveness of PRF and conventional blood clot scaffolds in RET. 

## Materials and methods

Study design and setting

This prospective, non-randomized comparative clinical study was conducted at the Department of Pediatric Dentistry, Pacific Hospital, Udaipur, Rajasthan, India. Participant recruitment was performed between May 2024 and December 2024, and clinical follow-up continued until March 2026. Participants received regenerative endodontic therapy using either a blood clot or PRF scaffold based on shared decision-making between the clinician and the parent or legal guardian. The study protocol was approved by the Institutional Ethics Committee (Approval No. PMU/PMCH/IEC/GEN/2024/144; approved on 12/04/2024) and conducted in accordance with the Declaration of Helsinki (2013). Written informed consent was obtained from parents or legal guardians, and assent was obtained from children when appropriate. This manuscript was prepared and reported in accordance with the Strengthening the Reporting of Observational Studies in Epidemiology (STROBE) guidelines for observational studies.

Participants

Children aged 7-16 years with non-vital immature permanent maxillary central incisors were consecutively screened. Pulp necrosis was confirmed by negative responses to cold testing (Endo-Frost; Coltene/Whaledent AG, Altstätten, Switzerland) and electric pulp testing (Digitest; Parkell Inc., Edgewood, NY, USA) [1]. Root immaturity (open apex >1 mm with thin dentinal walls) was verified on CBCT (Planmeca ProMax® 3D Mid; Planmeca Oy, Helsinki, Finland) using standardized acquisition parameters (120 kVp, 3-7 mA, 0.3 mm voxel size, 9 s exposure) following the ALARA principle [1]. CBCT examinations were performed at baseline and the 15-month follow-up.

Inclusion Criteria

The inclusion criteria were children aged 7-16 years; non-vital immature permanent maxillary central incisors; an open apex >1 mm with thin dentinal walls; chronic apical periodontitis; a restorable crown; no previous RET; the ability to attend scheduled follow-up visits; and written informed consent from a parent or legal guardian, with child assent obtained when appropriate.

Exclusion Criteria

The exclusion criteria were root resorption or root fracture; periodontal disease (probing depth >3 mm); non-restorable teeth; developmental dental anomalies; systemic conditions affecting healing; immunosuppression; antibiotic use within the previous three months; and allergy to study materials.

Sample size

Sample size was estimated using G*Power version 3.1.9.7 (Heinrich Heine University Düsseldorf, Germany) for a two-tailed comparison of two independent means, based on effect estimates derived from previously published CBCT-based regenerative endodontic studies [1]. An anticipated large effect size (Cohen's d = 0.90) was used for the sample size calculation.

Assuming α = 0.05, 80% power, and a 20% attrition rate, 20 teeth were required per group (total n = 40).

Allocation and blinding

Randomization was not feasible because PRF preparation requires venipuncture, which some children may refuse. Therefore, allocation was based on shared decision-making after standardized verbal explanation of both scaffold techniques, including the procedure, expected benefits, potential risks, and the requirement for venous blood collection for PRF. The final treatment decision reflected parental preference and the child's willingness to undergo venipuncture rather than clinician preference. Owing to the non-randomized design, adjustment for potential confounders was prespecified in the statistical analysis.

The treating clinician could not be blinded. However, two calibrated endodontists blinded to treatment allocation independently assessed all radiographic images. Calibration using 20 CBCT scans not included in the final analysis demonstrated excellent inter- and intra-examiner reliability (ICC = 0.94 and 0.96, respectively) [1].

Treatment protocol

All procedures were performed by a single trained clinician under rubber dam isolation (Hygenic® Dental Dam; Coltene/Whaledent AG, Altstätten, Switzerland) following the American Association of Endodontists guidelines [11].

First Appointment

Following local anesthesia (4% articaine with 1:100,000 epinephrine), access cavity preparation was performed, and working length was determined using a Root ZX II electronic apex locator (J. Morita Corp., Kyoto, Japan) with radiographic confirmation. Minimal instrumentation was performed using H-files (Mani Inc., Tochigi, Japan) to preserve dentinal walls [3,11]. Canal disinfection consisted of passive irrigation with 20 mL of 1.5% sodium hypochlorite delivered over 5 min, followed by 20 mL saline and 20 mL of 17% EDTA over 1 min using a 30-gauge side-vented needle (CanalClean; BioDent, Paju, Republic of Korea) positioned 2 mm short of the working length [11,12]. Calcium hydroxide paste (Metapaste; Meta Biomed Co., Ltd., Cheongju, Republic of Korea) was placed as the intracanal medicament, and the access cavity was sealed with resin-modified glass ionomer (Fuji II LC; GC Corporation, Tokyo, Japan). Patients were recalled after two to four weeks [11].

Second Appointment

Asymptomatic teeth underwent regenerative therapy after repeating the irrigation protocol. In the blood clot group, bleeding was induced by advancing a pre-curved #20 K-file 2 mm beyond the apex under 2% lidocaine without vasoconstrictor until a clot formed 2-3 mm below the cementoenamel junction [1]. In the PRF group, 10 mL of venous blood was centrifuged at 3000 rpm for 10 min using a REMI R-8C centrifuge (Remi Elektrotechnik Ltd., Mumbai, India) according to Choukroun's technique [13]. The PRF clot was sectioned and condensed into the canal using endodontic pluggers [1].

In both groups, a 2-3 mm layer of Bio MTA (Cerkamed Medical Company, Stalowa Wola, Poland) was placed over the scaffold, followed by restoration with resin-modified glass ionomer and composite resin (Neo Spectra ST; Dentsply Sirona, Konstanz, Germany) [1].

Outcome assessment

Clinical evaluations were performed at 3, 6, 9, 12, and 15 months by a blinded investigator using the criteria described by Johns et al. [14], including mobility, swelling or sinus tract, and tenderness to percussion and palpation. CBCT examinations were performed only when clinically indicated for diagnosis and treatment planning at baseline and the 15-month follow-up; the study utilized these clinically acquired scans without additional radiation exposure solely for research purposes. Periapical radiographs were obtained at every follow-up visit. No interim analyses were performed.

The prespecified primary outcome was the percentage increase in RRA at 15 months [15]. Secondary outcomes included percentage changes in root length, apical diameter, and periapical lesion size. CBCT measurements were performed using Romexis® software version 6.2 (Planmeca Oy, Helsinki, Finland) by two calibrated, blinded examiners, and the mean of both measurements was used for analysis [1,15].

Clinical success was defined as the absence of spontaneous pain, tenderness to percussion or palpation, swelling, sinus tract, abnormal mobility, and functional discomfort at follow-up [1]. Radiographic success was defined as continued root maturation, evidenced by increased root length, dentinal wall thickening, progressive apical closure, and reduction or complete resolution of the periapical radiolucency [1,15].

Statistical analysis

Statistical analyses were performed using IBM SPSS Statistics for Windows, Version 29.0 (Released 2022; IBM Corp., Armonk, NY, USA). Statistical graphics were generated using RStudio version 2026.06.0+242 (Posit Software, Boston, MA, USA), and study diagrams were prepared using diagrams.net. All tests were two-sided. Continuous variables are presented as mean ± standard deviation (SD) or median (interquartile range (IQR)), as appropriate. Normality and homogeneity of variance were assessed using the Shapiro-Wilk and Levene tests, respectively. Baseline comparisons were performed using the independent-samples t test or Mann-Whitney U test for continuous variables and the chi-square or Fisher's exact test for categorical variables.

The prespecified primary outcome was analyzed using multivariable linear regression adjusted for age, sex, trauma history, baseline root length, baseline apical diameter, baseline RRA, and baseline periapical lesion size. Covariates were selected a priori based on clinical relevance. Regression coefficients, 95% confidence intervals (CIs), and p-values were reported. Model assumptions were evaluated using residual and normal Q-Q plots, and multicollinearity was assessed using variance inflation factors (VIFs), with values <5 considered acceptable.

Secondary outcomes were analyzed using appropriate parametric or non-parametric tests. Longitudinal clinical outcomes were analyzed using linear mixed-effects models with treatment group, time, and group × time interaction as fixed effects and participant as a random intercept. Clinical success was compared using the chi-square or Fisher's exact test, as appropriate. Risk ratios with 95% CIs were calculated using the Wald log-transformation method. Effect sizes (Cohen's d and Cramer's V) were reported. Bonferroni correction was applied for multiple comparisons. Participants with missing primary outcome data (n = 3, 7.5%) were excluded using a complete-case approach because of the low proportion of missing data; therefore, multiple imputation was not performed. Sensitivity analyses excluding protocol deviations yielded similar findings. A two-sided p-value <0.05 was considered statistically significant.

## Results

Study population and baseline characteristics

**Figure 1:**
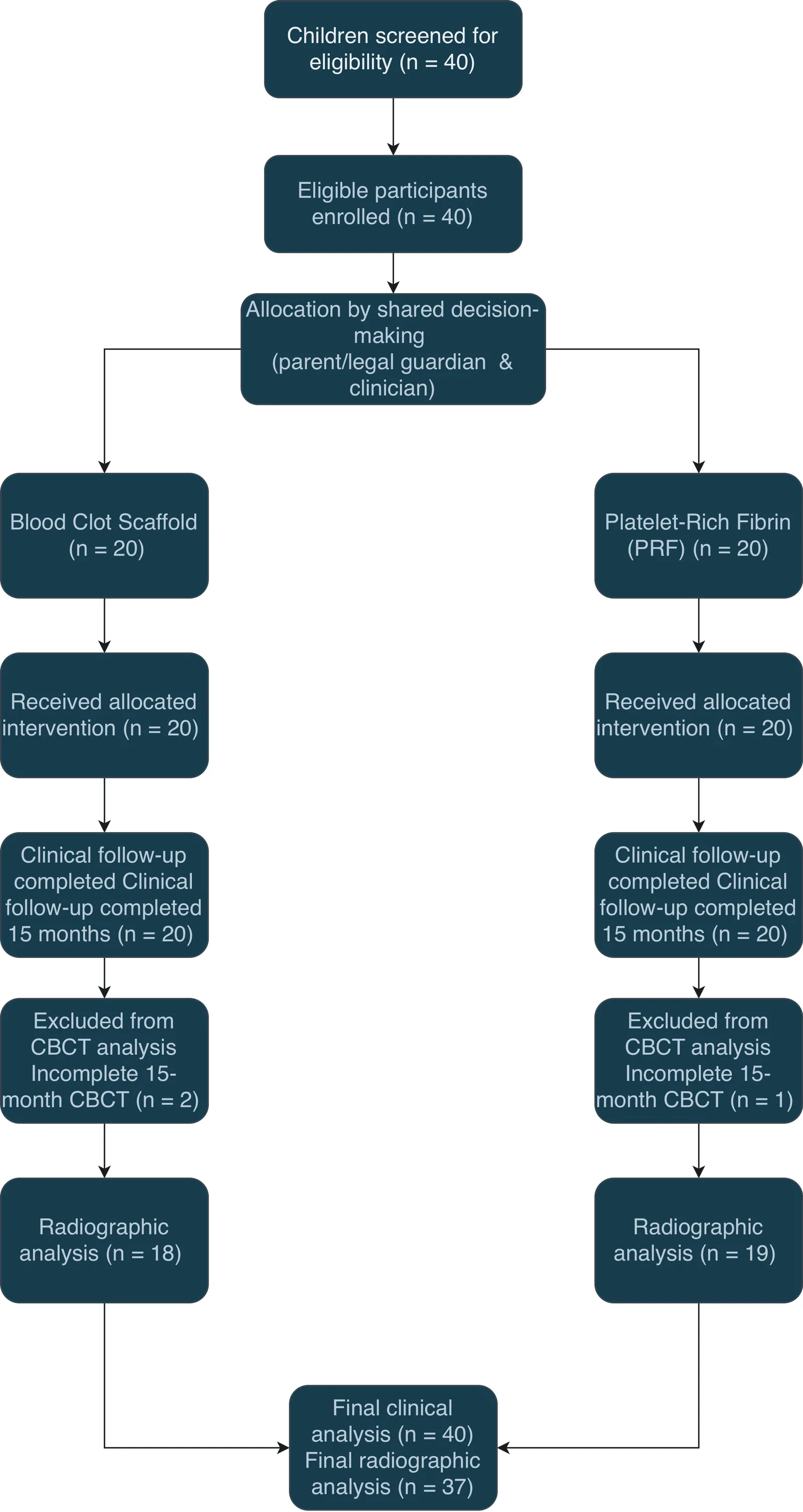
Study flow diagram Study flow diagram illustrating participant recruitment, allocation through shared decision-making, follow-up, and inclusion in the final clinical and radiographic analyses. Three participants (two in the Blood Clot group and one in the Platelet-Rich Fibrin (PRF) group) were excluded from the radiographic analysis because of incomplete 15-month cone-beam computed tomography (CBCT) imaging. Clinical analysis included all 40 participants, whereas radiographic analysis included 37 participants.

A total of 40 patients (20 in the blood clot group and 20 in the PRF group) were included in the clinical analysis. Three participants (two in the blood clot group and one in the PRF group) were excluded from the radiographic analysis due to incomplete CBCT imaging at the 15-month follow-up, resulting in a radiographic sample of 37 teeth (18 in the blood clot group and 19 in the PRF group). The participant flow diagram is presented in Figure 1.

The baseline demographic and clinical characteristics of both groups are presented in Table 1. There were no statistically significant differences between the two groups at baseline for any parameter, confirming the comparability of the groups. The mean age was 10.45 ± 2.31 years in the blood clot group and 10.85 ± 2.52 years in the PRF group (p = 0.605). Both groups had a slight female predominance (60% vs. 50%, p = 0.519). The majority of teeth treated were maxillary central incisors (85% in the blood clot group and 85% in the PRF group), and a history of dental trauma was present in 75% and 70% of cases, respectively (p = 0.724).

**Table 1:** Baseline demographic and clinical characteristics of study participants Independent samples t-test was used for continuous variables, and a chi-square test was used for categorical variables. All p-values are two-sided, and p < 0.05 was considered statistically significant. Notes: Clinical analysis included all 40 participants. Radiographic analyses excluded three participants with incomplete CBCT. AbPRF, Platelet-Rich Fibrin; SD, Standard Deviation; CBCT, Cone-Beam Computed Tomography

Characteristic	Blood Clot Group (n = 20)	PRF Group (n = 20)	Test Statistic	p-value	Effect Size
Age (years), Mean ± SD	10.45 ± 2.31	10.85 ± 2.52	t(38) = 0.521	0.605	Cohen's d = 0.165
Sex, n (%)	-	-	χ²(1) = 0.417	0.519	Cramer's V = 0.102
Male	8 (40.0%)	10 (50.0%)	-	-	-
Female	12 (60.0%)	10 (50.0%)	-	-	-
Tooth Type, n (%)	-	-	χ²(2) = 0.225	0.893	Cramer's V = 0.075
Maxillary Central Incisor (#11)	8 (40.0%)	7 (35.0%)	-	-	-
Maxillary Central Incisor (#21)	9 (45.0%)	10 (50.0%)	-	-	-
Other Permanent Incisors (Maxillary Lateral or Mandibular Incisors)	3 (15.0%)	3 (15.0%)	-	-	-
History of Dental Trauma, n (%)	15 (75.0%)	14 (70.0%)	χ²(1) = 0.125	0.724	Cramer's V = 0.056
Baseline Clinical Parameters	-	-	-	-	-
Spontaneous Pain, n (%)	11 (55.0%)	9 (45.0%)	χ²(1) = 0.400	0.527	Cramer's V = 0.100
Swelling, n (%)	5 (25.0%)	6 (30.0%)	χ²(1) = 0.125	0.724	Cramer's V = 0.056
Sinus Tract, n (%)	2 (10.0%)	3 (15.0%)	χ²(1) = 0.229	0.633	Cramer's V = 0.076
Tenderness to Percussion, n (%)	7 (35.0%)	5 (25.0%)	χ²(1) = 0.476	0.490	Cramer's V = 0.109
Tenderness to Palpation, n (%)	4 (20.0%)	6 (30.0%)	χ²(1) = 0.533	0.465	Cramer's V = 0.115
Tooth Mobility, n (%)	3 (15.0%)	2 (10.0%)	χ²(1) = 0.229	0.633	Cramer's V = 0.076
Baseline Radiographic Parameters, Mean ± SD	-	-	-	-	-
Root Length (mm)	12.38 ± 1.65	12.51 ± 1.72	t(38) = 0.244	0.809	Cohen's d = 0.077
Apical Diameter (mm)	1.48 ± 0.42	1.52 ± 0.44	t(38) = 0.293	0.771	Cohen's d = 0.093
Radiographic Root Area (mm²)	37.12 ± 6.85	36.85 ± 7.12	t(38) = 0.122	0.904	Cohen's d = 0.039
Periapical Lesion Size (mm²)	26.45 ± 8.12	27.15 ± 9.23	t(38) = 0.249	0.805	Cohen's d = 0.079
Follow-up Duration (months), Mean ± SD	14.85 ± 0.89	14.65 ± 1.23	t(38) = 0.593	0.557	Cohen's d = 0.188

Baseline clinical parameters, including spontaneous pain, swelling, sinus tract, tenderness to percussion and palpation, and tooth mobility, showed no significant differences between groups (all p > 0.05). Similarly, baseline radiographic parameters, including root length (p = 0.809), apical diameter (p = 0.771), RRA (p = 0.904), and periapical lesion size (p = 0.805), were comparable between groups. The mean follow-up duration was 14.85 ± 0.89 months and 14.65 ± 1.23 months for the blood clot and PRF groups, respectively (p = 0.557).

Primary outcome: percentage increase in RRA

**Figure 2:**
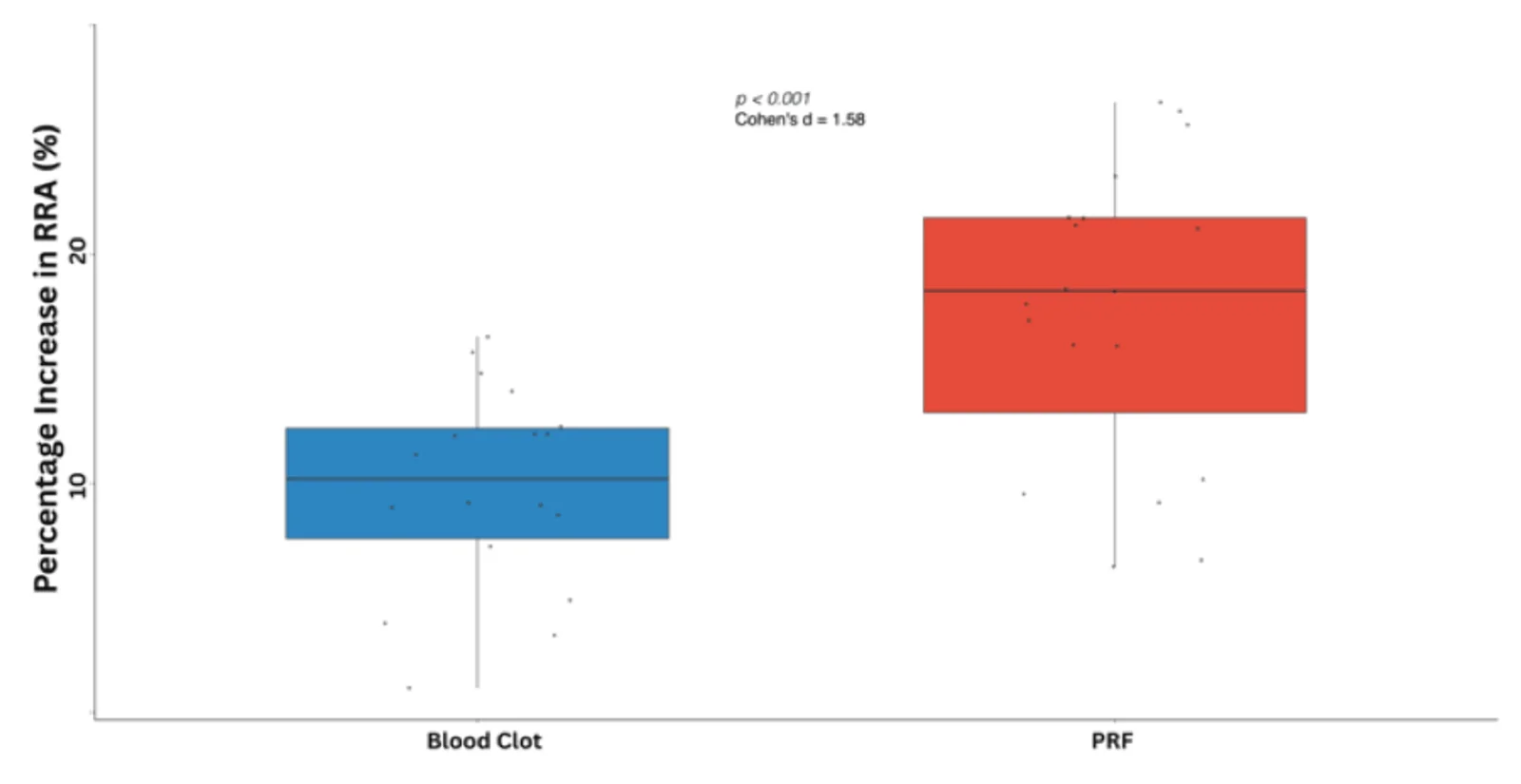
Boxplot comparing the percentage increase in RRA Boxplot comparing the percentage increase in RRA from baseline to the 15-month follow-up in the Blood Clot (n = 18) and PRF (n = 19) groups. The central line represents the median, boxes represent the interquartile range (IQR), and whiskers extend to 1.5 × IQR. Points beyond the whiskers represent outliers. PRF, Platelet-Rich Fibrin; RRA, Radiographic Root Area

The primary radiographic outcome is summarized in Table 1 and illustrated in Figure 2. The PRF group demonstrated a significantly greater percentage increase in RRA than the blood clot group (19.15 ± 6.22% vs. 9.72 ± 5.18%; mean difference, 9.43%; 95% CI, 5.86-13.00; p < 0.001; Cohen's d = 1.58) (Table 2 and Figure 2).

**Table 2:** Primary and secondary radiographic outcomes at 15-month follow-up † Independent samples t-test; *p < 0.05 was considered statistically significant. Between-group comparison of absolute 15-month values was exploratory; the prespecified primary endpoint was percentage change from baseline. Notes: Complete-case analysis (n = 37); Three participants were excluded due to incomplete CBCT at 15 months (two in Blood Clot and one in PRF). Data expressed as Mean ± SD. RRA, Radiographic Root Area; PRF, Platelet-Rich Fibrin; SD, Standard Deviation; CI, Confidence Interval

Parameter	Blood Clot Group (n = 18)	PRF Group (n = 19)	Mean Difference (95% CI)	p-value	Effect Size (Cohen's d)
Primary Outcome	-	-	-	-	-
% Increase in RRA	9.72 ± 5.18	19.15 ± 6.22	9.43 (5.86, 13.00)	<0.001*	1.58
Secondary Outcomes	-	-	-	-	-
% Increase in Root Length	5.18 ± 2.85	5.98 ± 3.08	0.80 (-1.15, 2.75)	0.415	0.27
% Reduction in Apical Diameter	29.72 ± 11.25	38.42 ± 12.61	8.70 (1.01, 16.39)	0.028*	0.73
% Reduction in Lesion Size	42.0 ± 18.28	68.0 ± 20.31	26.0 (14.0, 38.0)	<0.001*	1.31
Absolute Changes (Exploratory)†	-	-	-	-	-
Root Length (mm)	12.38 → 13.03	12.51 → 13.25	0.09 (-0.42, 0.60)	0.723	-
Apical Diameter (mm)	1.48 → 1.04	1.52 → 0.94	0.10 (-0.08, 0.28)	0.269	-
RRA (mm²)	37.12 → 40.78	36.85 → 43.82	3.04 (-0.89, 6.97)	0.126	-
Lesion Size (mm²)	26.45 → 15.34	27.15 → 8.69	6.65 (0.89, 12.41)	0.024*	-

Secondary radiographic outcomes

Secondary radiographic outcomes included percentage increase in root length, percentage reduction in apical diameter, and percentage reduction in periapical lesion size. The results are summarized in Table 2.

Root Length

The PRF group showed a slightly greater percentage increase in root length than the blood clot group (5.98 ± 3.08% vs. 5.18 ± 2.85%); however, this difference was not statistically significant (mean difference, 0.80%; 95% CI, -1.15 to 2.75; p = 0.415) and was associated with a small effect size (Cohen's d = 0.27; Table 2).

Apical Diameter

The PRF group demonstrated a significantly greater percentage reduction in apical diameter (38.42 ± 12.61%) compared to the blood clot group (29.72 ± 11.25%; mean difference, 8.70%; 95% CI, 1.01 to 16.39; p = 0.028; Cohen's d = 0.73; Table 2).

Periapical Lesion Size

**Figure 3:**
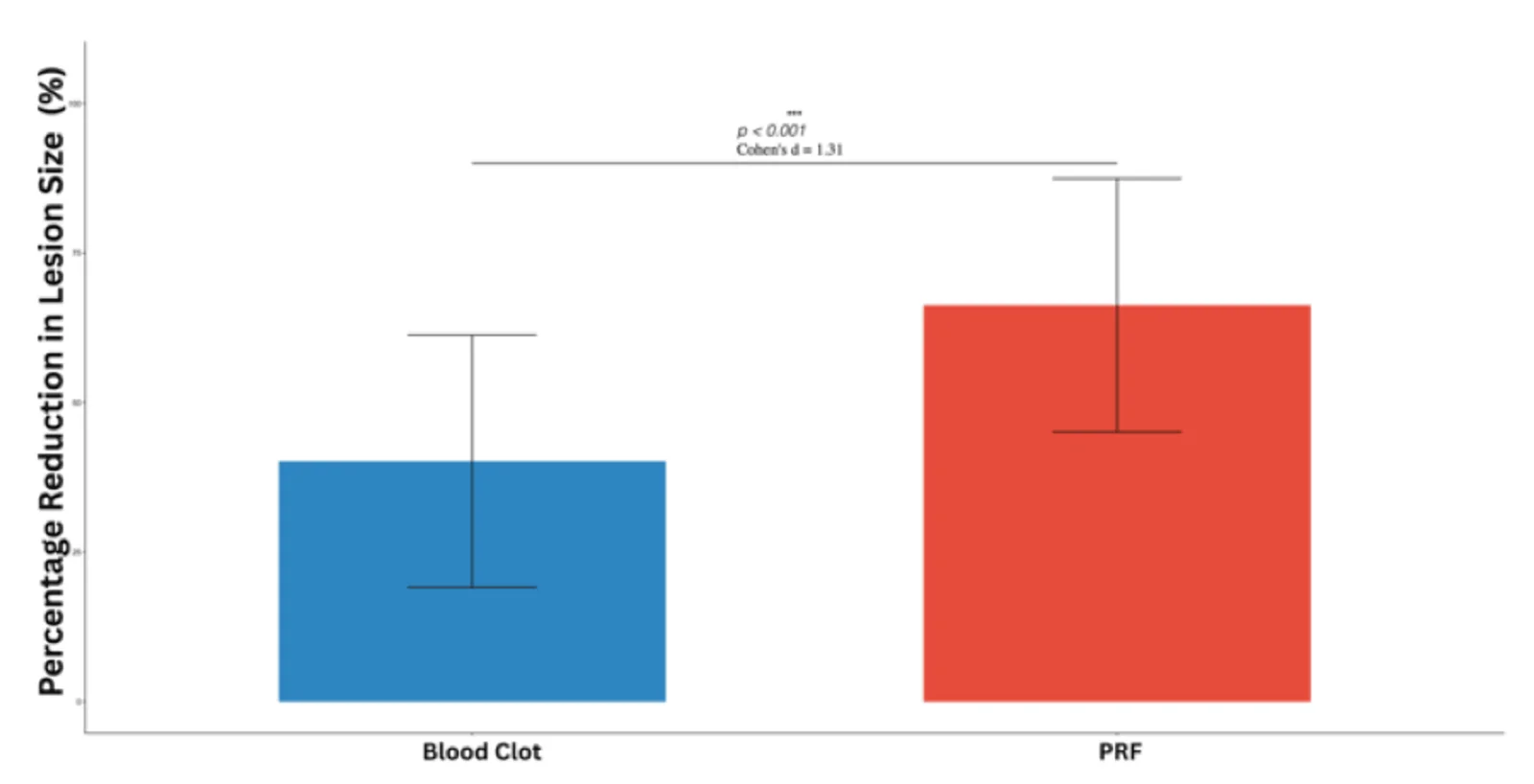
Percentage reduction in periapical lesion size Percentage reduction in periapical lesion size in Blood Clot (n = 18) and PRF (n = 19) groups at 15-month follow-up. Bars represent mean values. Error bars represent standard deviation. Statistical test: Independent-samples t-test; mean difference = 26.0% (95% CI, 14.0-38.0); p < 0.001; Cohen's d = 1.31 (large effect). ***p < 0.001 indicates a statistically significant difference between groups. PRF, Platelet-Rich Fibrin; SD, Standard Deviation

The PRF group showed a significantly greater percentage reduction in lesion size (68.0 ± 20.31%) compared to the blood clot group (42.0 ± 18.28%; mean difference, 26.0%; 95% CI, 14.0 to 38.0; p < 0.001; Cohen's d = 1.31; Table 1 and Figure 3).

Within-group changes

To evaluate the effectiveness of each treatment modality independently, within-group changes from baseline to 15 months were analyzed using paired t-tests. The within-group changes are summarized in Table 3.

**Table 3:** Within-group changes in radiographic parameters from baseline to 15 months † Paired t-test; *p < 0.05 considered statistically significant. Note: Complete-case analysis (n = 37); Data expressed as Mean ± SD. PRF, Platelet-Rich Fibrin; SD, Standard Deviation

Parameter	Blood Clot Group (n = 18)			PRF Group (n = 19)		
	Baseline	15 Months	p-value†	Baseline	15 Months	p-value†
Root Length (mm)	12.38 ± 1.65	13.03 ± 1.58	0.012*	12.51 ± 1.72	13.25 ± 1.65	0.009*
Apical Diameter (mm)	1.48 ± 0.42	1.04 ± 0.35	0.004*	1.52 ± 0.44	0.94 ± 0.31	0.002*
Radiographic Root Area (mm²)	37.12 ± 6.85	40.78 ± 6.52	0.018*	36.85 ± 7.12	43.82 ± 6.85	<0.001*
Periapical Lesion Size (mm²)	26.45 ± 8.12	15.34 ± 6.85	<0.001*	27.15 ± 9.23	8.69 ± 5.42	<0.001*

Blood Clot Group

Significant improvements were observed in all radiographic parameters. Root length increased from 12.38 ± 1.65 mm to 13.03 ± 1.58 mm (p = 0.012). Apical diameter decreased from 1.48 ± 0.42 mm to 1.04 ± 0.35 mm (p = 0.004). RRA increased from 37.12 ± 6.85 mm² to 40.78 ± 6.52 mm² (p = 0.018). Periapical lesion size decreased from 26.45 ± 8.12 mm² to 15.34 ± 6.85 mm² (p < 0.001).

PRF Group

Similarly, significant improvements were observed in all radiographic parameters. Root length increased from 12.51 ± 1.72 mm to 13.25 ± 1.65 mm (p = 0.009). Apical diameter decreased from 1.52 ± 0.44 mm to 0.94 ± 0.31 mm (p = 0.002). RRA increased from 36.85 ± 7.12 mm² to 43.82 ± 6.85 mm² (p < 0.001). Periapical lesion size decreased from 27.15 ± 9.23 mm² to 8.69 ± 5.42 mm² (p < 0.001).

Clinical success outcomes

The clinical success outcomes at the 15-month follow-up are summarized in Table 4. The PRF group showed slightly higher success rates for all parameters, but no statistically significant differences were observed.

**Table 4:** Clinical success outcomes at 15-month follow-up †Fisher's exact test; p < 0.05 considered statistically significant. The 95% confidence interval for the risk ratio was estimated using the Wald log-transformation method, whereas statistical significance was assessed using Fisher's exact test because of the small sample size and the presence of a zero-event cell in the PRF group. Notes: Clinical outcomes based on all 40 participants. Radiographic success based on 37 participants with complete CBCT. Based on AAE guidelines [1] and Flake et al. [10]. PRF, Platelet-Rich Fibrin; CI, Confidence Interval

Parameter	Blood Clot Group (n = 20)	PRF Group (n = 20)	Risk Ratio (95% CI)	p-value†	Effect Size
Clinical Success Rate	-	-	-	-	-
Resolution of Spontaneous Pain	19 (95.0%)	20 (100%)	1.05 (0.92, 1.21)	0.317	Cramer's V = 0.160
Resolution of Swelling	18 (90.0%)	20 (100%)	1.11 (0.95, 1.29)	0.147	Cramer's V = 0.229
Resolution of Sinus Tract	19 (95.0%)	20 (100%)	1.05 (0.92, 1.21)	0.317	Cramer's V = 0.160
Resolution of Tenderness to Percussion	17 (85.0%)	19 (95.0%)	1.12 (0.91, 1.37)	0.274	Cramer's V = 0.174
Resolution of Tenderness to Palpation	19 (95.0%)	20 (100%)	1.05 (0.92, 1.21)	0.317	Cramer's V = 0.160
Resolution of Abnormal Mobility	18 (90.0%)	18 (90.0%)	1.00 (0.81, 1.24)	>0.999	Cramer's V = 0.000
Overall Clinical Success	18 (90.0%)	19 (95.0%)	1.06 (0.88, 1.28)	0.548	Cramer's V = 0.095
Radiographic Success (n = 37)	15/18 (83.3%)	19/19 (100%)	1.20 (1.01, 1.42)	0.073	Cramer's V = 0.285

Overall Clinical Success

Overall clinical success was achieved by 90% (18/20) of participants in the blood clot group and 95% (19/20) of participants in the PRF group (p = 0.548; Cramer's V = 0.095).

Radiographic Success

In the radiographic analysis (n = 37), 83.3% (15/18) of participants in the blood clot group and 100% (19/19) of participants in the PRF group achieved radiographic success (risk ratio = 1.20; 95% CI, 1.01-1.42; Fisher's exact test, p = 0.073; Cramer's V = 0.285).

Longitudinal clinical outcomes

**Figure 4:**
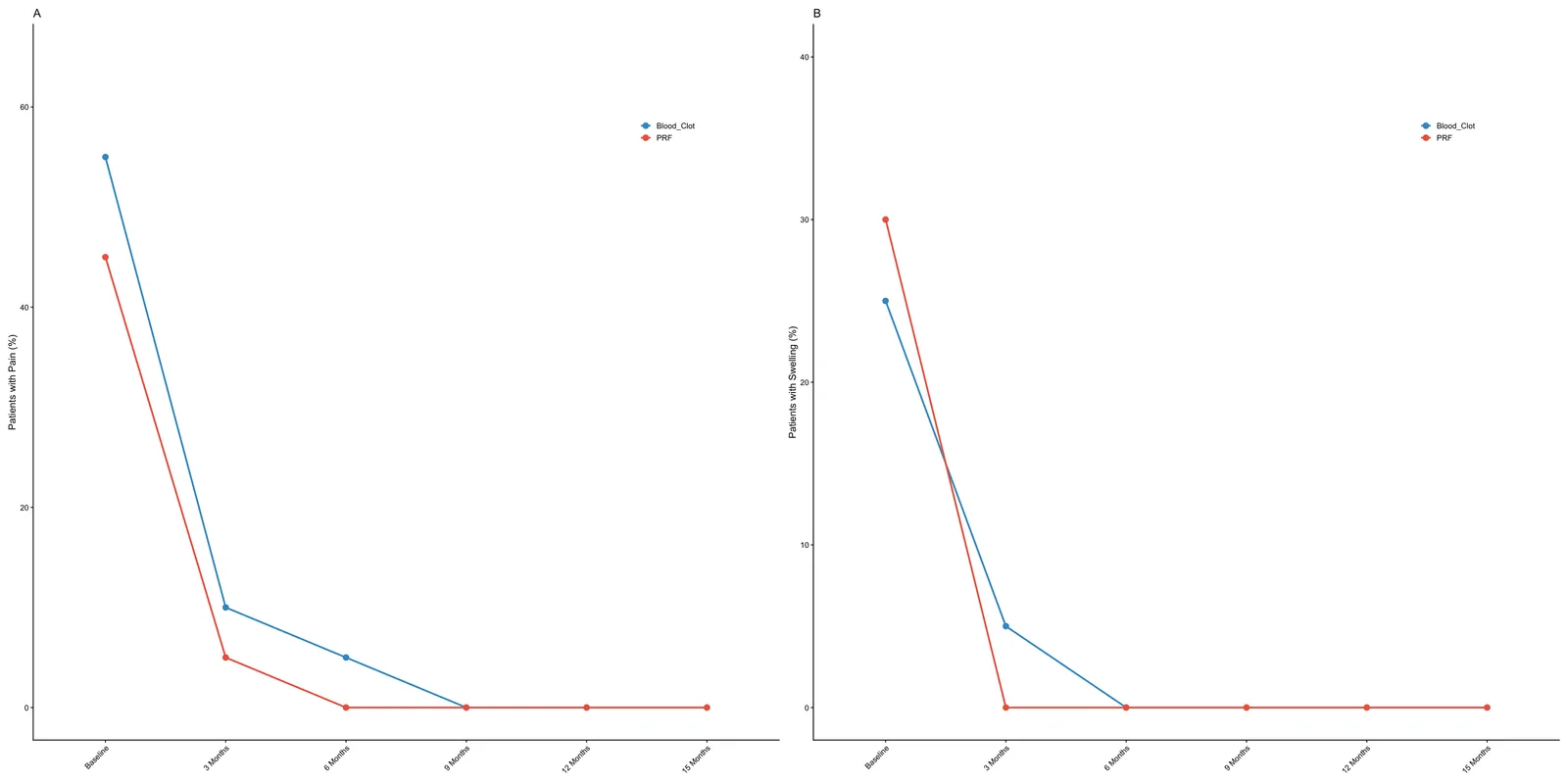
Longitudinal changes in clinical symptoms - pain and swelling Longitudinal changes in clinical symptoms over the 15-month follow-up: (A) Pain resolution; (B) Swelling resolution. Statistical test: Fisher's exact test; p < 0.05 was considered statistically significant.

Clinical outcomes were assessed at multiple time points (baseline, 3, 6, 9, 12, and 15 months). The results are presented in Table 5 and illustrated in Figure 4.

**Table 5:** Longitudinal clinical outcomes over 15-month follow-up Fisher's exact test; p < 0.05 considered statistically significant. Note: Clinical outcomes based on all 40 participants.

Time Point	Blood Clot Group (n = 20)		PRF Group (n = 20)		Between-Group Difference	
	Pain Present, n (%)	Swelling Present, n (%)	Pain Present, n (%)	Swelling Present, n (%)	p-value (Pain)	p-value (Swelling)
Baseline	11 (55.0%)	5 (25.0%)	9 (45.0%)	6 (30.0%)	0.527	0.724
3 Months	2 (10.0%)	1 (5.0%)	1 (5.0%)	0 (0.0%)	0.548	0.317
6 Months	1 (5.0%)	0 (0.0%)	0 (0.0%)	0 (0.0%)	0.317	>0.999
9 Months	0 (0.0%)	0 (0.0%)	0 (0.0%)	0 (0.0%)	>0.999	>0.999
12 Months	0 (0.0%)	0 (0.0%)	0 (0.0%)	0 (0.0%)	>0.999	>0.999
15 Months	0 (0.0%)	0 (0.0%)	0 (0.0%)	0 (0.0%)	>0.999	>0.999

At baseline, spontaneous pain was present in 55% of the blood clot group and 45% of the PRF group (p = 0.527). Swelling was present in 25% and 30% of cases, respectively (p = 0.724). By three months, pain had resolved in the majority of patients, with only 10% (2/20) in the blood clot group and 5% (1/20) in the PRF group reporting pain (p = 0.548). At six months, only one patient (5%) in the blood clot group reported pain, while no patients in the PRF group reported pain (p = 0.317). By nine months and at all subsequent time points, no patients in either group reported pain or swelling.

Resolution of other clinical parameters, including sinus tract, tenderness to percussion, tenderness to palpation, and abnormal mobility, was comparable between groups, with no statistically significant differences at any time point.

Multivariable regression analysis

A multivariable linear regression analysis was performed to identify factors independently associated with the primary outcome (percentage increase in RRA). The model included scaffold type, age, sex, history of trauma, baseline root length, baseline apical diameter, baseline RRA, and baseline lesion size. The multivariable regression results are presented in Table 6.

**Table 6:** Multivariable linear regression analysis for primary outcome (percentage increase in RRA) Model R² = 0.53; Adjusted R² = 0.47; F(8,28) = 3.95; p < 0.001; Durbin-Watson = 1.92. *Multivariable linear regression with complete-case analysis (n = 37); p < 0.05 considered statistically significant. RRA, Radiographic Root Area; PRF, Platelet-Rich Fibrin; VIF, Variance Inflation Factor; CI, Confidence Interval

Variable	β Coefficient	95% CI	Standardized β	p-value	VIF
Scaffold Type (PRF vs. Blood Clot)	9.43	5.86, 13.00	0.51	<0.001*	1.19
Age (years)	-0.42	-1.09, 0.25	-0.13	0.214	1.07
Sex (Female vs. Male)	1.52	-2.21, 5.25	0.08	0.416	1.04
History of Trauma	-2.08	-6.41, 2.25	-0.10	0.338	1.11
Baseline Root Length (mm)	-0.81	-1.91, 0.29	-0.15	0.144	1.14
Baseline Apical Diameter (mm)	-3.42	-8.09, 1.25	-0.15	0.147	1.17
Baseline RRA (mm²)	-0.30	-0.66, 0.06	-0.20	0.098	1.24
Baseline Lesion Size (mm²)	-0.27	-0.54, -0.00	-0.24	0.045*	1.09

The model was statistically significant (F(8,28) = 3.95; p < 0.001) and explained 53% of the variance in the primary outcome (R² = 0.53; adjusted R² = 0.47). The Durbin-Watson statistic was 1.92, indicating no significant autocorrelation.

Scaffold type (PRF vs. blood clot) was the strongest independent predictor of percentage increase in RRA (β = 9.43; 95% CI, 5.86 to 13.00; standardized β = 0.51; p < 0.001). Baseline lesion size was also a significant predictor (β = -0.27; 95% CI, -0.54 to -0.00; standardized β = -0.24; p = 0.045), indicating that smaller baseline lesions were associated with a greater percentage increase in RRA.

Other variables, including age (p = 0.214), sex (p = 0.416), history of trauma (p = 0.338), baseline root length (p = 0.144), baseline apical diameter (p = 0.147), and baseline RRA (p = 0.098), were not significant independent predictors. The VIF values ranged from 1.04 to 1.24, indicating no significant multicollinearity.

## Discussion

These findings were supported by significantly greater percentage increases in RRA and greater reductions in apical diameter and periapical lesion size (Table 3). PRF achieved a significantly greater percentage increase in RRA, greater reduction in apical diameter, and more pronounced periapical lesion healing, whereas both treatment modalities achieved similarly high clinical success rates. Multivariable regression further identified scaffold type as the strongest independent predictor of radiographic root development, emphasizing the importance of scaffold selection in regenerative endodontics. These findings support the biological principles of regenerative therapy, whereby successful regeneration depends on effective canal disinfection, stem cell recruitment, signaling molecules, and an appropriate biological scaffold [1-3].

The superior radiographic outcomes observed with PRF are biologically plausible. PRF provides a dense fibrin matrix capable of sustained release of platelet-derived growth factor, transforming growth factor-β, vascular endothelial growth factor, and other cytokines that enhance angiogenesis, stem cell migration, extracellular matrix deposition, and tissue remodeling [4,12,13]. Furthermore, appropriate dentin conditioning promotes the release of bioactive dentin-derived growth factors, creating a favorable microenvironment for odontoblastic differentiation and continued root maturation [5]. These biological mechanisms likely contributed to the significantly greater increase in RRA and enhanced periapical healing observed in the PRF group.

The present findings are consistent with recent comparative clinical investigations demonstrating superior radiographic outcomes with PRF-based regenerative procedures compared with conventional blood clot scaffolds [6,16,17]. A prospective clinical study reported significant improvements in root thickness and periapical healing following the use of PRF-based scaffolds in traumatized immature permanent teeth [6]. Similarly, a randomized clinical trial demonstrated that PRF promoted favorable radiographic root maturation and greater reduction in periapical lesion size when compared with another biologically active scaffold [7]. Randomized clinical evidence has shown that biologically active scaffolds improve root maturation and periapical healing, supporting the concept that scaffold composition influences regenerative outcomes beyond infection control alone [7,18-20]. More importantly, systematic reviews of randomized clinical trials and recent meta-analyses have concluded that platelet concentrate scaffolds generally provide superior radiographic root development and periapical healing than conventional blood clot scaffolds, although heterogeneity among treatment protocols and outcome measures continues to limit the certainty of the available evidence [8,9,18,19,21]. A retrospective comparative study similarly reported favorable clinical and radiographic outcomes with both PRF and blood clot scaffolds, with PRF showing numerically greater root development [16]. The present study strengthens this evidence by incorporating standardized CBCT-based quantitative assessment and objective measurement of RRA.

An important observation was that PRF significantly improved RRA and periapical healing without producing a significant additional increase in root length. This finding suggests that the principal regenerative advantage of PRF may arise from enhanced dentinal wall thickening and apical maturation rather than accelerated longitudinal root elongation. Because RRA integrates changes in both root length and dentinal wall thickness into a single quantitative measurement, it provides a more comprehensive assessment of root maturation than isolated linear parameters [10,15,22]. The use of CBCT-based quantitative assessment represents a methodological strength by enabling reproducible three-dimensional evaluation of root morphology and periapical healing, overcoming the geometric distortion and dimensional limitations inherent to conventional two-dimensional periapical radiography [10,23]. These findings are consistent with a recent randomized clinical trial demonstrating good agreement between CBCT and periapical radiography for quantitative assessment of regenerative endodontic outcomes [20]. Furthermore, regression analysis demonstrated that scaffold type remained an independent determinant of regenerative success after adjustment for baseline demographic and radiographic variables, while larger baseline periapical lesions were associated with comparatively smaller improvements in RRA.

Although PRF demonstrated superior radiographic performance, both treatment groups achieved high clinical success, with complete resolution of pain and swelling during follow-up and no statistically significant differences in overall clinical success. These findings indicate that conventional blood clot scaffolds remain effective for eliminating clinical signs and symptoms, whereas PRF primarily provides additional benefits in structural root maturation, consistent with the American Association of Endodontists' clinical considerations, which identify elimination of symptoms and bony healing as the primary goal of regenerative procedures [1,24]. Clinically, enhanced root development may improve fracture resistance and long-term tooth retention in immature permanent teeth [21,24]. These findings may have important clinical implications by improving the long-term structural integrity of immature permanent teeth and potentially reducing the need for future endodontic or restorative interventions [25]. From a clinical perspective, PRF may be considered the preferred scaffold when venous blood collection is feasible, particularly for immature permanent teeth requiring enhanced structural root maturation. Conversely, conventional blood clot scaffolds remain an effective and practical alternative when PRF preparation is not feasible or is declined by the patient or parent because comparable clinical success was achieved despite less favorable radiographic maturation.

Recent randomized clinical trials, multicenter investigations, and systematic reviews continue to emphasize the need for standardized regenerative protocols, CBCT-based outcome assessment, larger sample sizes, and longer follow-up to strengthen the evidence base for scaffold selection in regenerative endodontics [20-25]. Randomized and multicenter clinical studies have demonstrated the feasibility of standardized CBCT-based quantitative assessment for evaluating regenerative outcomes while highlighting the importance of harmonized treatment protocols to improve comparability across studies [20,22,23]. Likewise, recent systematic reviews and network meta-analyses recommend standardized scaffold preparation techniques, uniform outcome measures, and adequately powered multicenter trials to establish the comparative effectiveness of platelet concentrates in regenerative endodontics [21,24].

## Conclusions

PRF significantly enhanced structural root maturation and periapical healing compared with the conventional blood clot scaffold while maintaining comparable clinical success. These findings suggest that PRF may provide additional benefits in promoting root maturation and long-term structural integrity in immature permanent teeth undergoing regenerative endodontic therapy. The integration of standardized CBCT-based quantitative assessment and RRA analysis provides robust evidence supporting PRF as an effective scaffold for RET and may facilitate evidence-based clinical decision-making. Further multicenter randomized controlled trials with larger sample sizes, standardized treatment protocols, and longer follow-up are warranted to confirm these findings and establish the long-term effectiveness of PRF-assisted regenerative endodontic therapy.
